# Honey Bee (Hymenoptera: Apidea) Pollen Forage in a Highly Cultivated Agroecosystem: Limited Diet Diversity and Its Relationship to Virus Resistance

**DOI:** 10.1093/jee/toaa055

**Published:** 2020-04-10

**Authors:** Ge Zhang, Ashley L St. Clair, Adam Dolezal, Amy L Toth, Matthew O’Neal

**Affiliations:** 1 Department of Entomology, Iowa State University, Ames, IA; 2 Department of Ecology, Evolution and Organismal Biology, Iowa State University, Ames, IA; 3 Department of Entomology, University of Illinois Urbana-Champaign, Urbana, IL

**Keywords:** soybean, legume, virus, honey bee, *Apis mellifera*

## Abstract

Intensified agriculture reduces natural and seminatural habitats and plant diversity, reducing forage available to honey bees (*Apis mellifera* L. [Hymenoptera: Apidea]). In agricultural landscapes of Iowa, United States, we studied the impact of extrinsic agricultural intensification on the availability of pollen for honey bees by placing colonies next to soybean fields surrounded by either a low or high level of cultivation. The abundance and diversity of pollen returned to a colony were estimated by placing pollen traps on bee colonies during the summer and fall of 2015 and 2016. We observed no difference in abundance and diversity of pollen collected by colonies in either landscape, but abundance varied over time with significantly less collected in September. We explored if the most commonly collected pollen from these landscapes had the capacity to support honey bee immune health by testing if diets consisting of these pollens improved bee resistance to a viral infection. Compared to bees denied pollen, a mixture of pollen from the two most common plant taxa (*Trifolium* spp. L. [Fabales: Fabaceae] and *Chimaechrista fasciculata* (Michx.) Greene [Fabales: Fabaceae]) significantly reduced honey bee mortality induced by viral infection. These data suggest that a community of a few common plants was favored by honey bees, and when available, could be valuable for reducing mortality from a viral infection. Our data suggest a late season shortage of pollen may be ameliorated by additions of fall flowering plants, like goldenrod (*Solidago* spp. L. [Asterales: Asteraceae]) and sunflower (*Helianthus*, *Heliopsis*, and *Silphium* spp. [Asterales: Asteraceae]), as options for enhancing pollen availability and quality for honey bees in agricultural landscapes.

Honey bees in the United States and Europe are exposed to multiple environmental stressors (Goulson et al. 2015), including a reduction in forage quantity and diversity (Vanengelsdorp and Meixner 2010). Intensified agriculture dramatically reduces the availability of natural and seminatural habitats, and is predicted to affect availability and diversity of forage across the entire growing season by decreasing overall plant diversity (Naug 2009). Pollen is a critical source of nutrition as it supplies proteins, lipids, and micronutrients (Wright et al. 2018). In agricultural landscapes with cropland as the dominant land cover, some mass-flowering crops, like sunflower and oilseed rape, can provide honey bees large quantities of pollen in a short period (Requier et al. 2015, Thom et al. 2018). However, a preponderance of only a single species could contribute to reduced nutritional value of pollen for honey bees (Schmidt 1984; Schmidt et al. 1987, 1995; Di Pasquale et al. 2013; Nicolson and Human 2013). After a crop blooms, honey bees may suffer from a shortage of forage in the surrounding landscapes due to limited natural or seminatural habitats.

The value of crop and noncrop habitat as a source of pollen forage for honey bees can be important to inform land and apiary management decisions. In previous studies, enhancements in pollen collection were not detected when honey bee colonies were placed in landscapes with varying amounts of noncrop habitats (Smart et al. 2016a,b; Danner et al. 2017). A general conclusion from these studies is that landscapes with less crop production do not necessarily result in an increase in the abundance and diversity of pollen collected by honey bees. One reason may be that these studies were performed in agricultural landscapes with a limited range of landscape diversity, resulting in reduced power to detect the effect of noncrop habitats on pollen forage (Danner et al. 2017). Another potential explanation may be the impact of the primary crop within these landscapes. Primary crops that are a source of pollen for honey bees may distract them from forage available in noncrop habitats (Danner et al. 2017). The response of honey bees to noncrop habitat may be stronger in landscapes where the crops are not a source of high-quality pollen for honey bees, such as soybean (*Glycine max* (L.) Merr. [Fabales: Fabaceae]) and corn (*Zea mays* L. [Poales: Poaceae]). Corn pollen is considered to be of limited nutritional value for honey bees (Höcherl et al. 2012) and less likely to be collected by honey bees. Honey bees can be found in soybean fields (Gill and O’Neal 2015) and collect nectar from soybean flowers (Villanueva-Gutiérrez et al. 2014, Wheelock et al. 2016, USDA 2017). However, soybean neither requires insect pollination, nor is it reported as a major pollen source for honey bees. Honey bees in a landscape dominated by these crops may focus more of their pollen foraging efforts in noncrop areas of a landscape rather than cultivated areas.

In this study, we predicted that honey bees kept in a landscape of low forage diversity and quality (i.e., where corn and soybean are extensively produced) would collect less abundant and a less diverse mixture of pollen, than those kept within a landscape with more noncrop habitat. We focused our study in the U.S. state of Iowa, where around 85% of the land is devoted to agriculture, and 64% of that land is used for corn and soybean production (USDA-NASS 2019). The intensive management of weeds in the Midwest has reduced floral diversity (Otto et al. 2016). To maximize our potential to observe honey bees using noncrop forage within this region, we selected soybean fields in locations that represented extremes of land use (i.e., landscapes that varied in the amount of farm cultivation as defined in Dolezal et al. (2019a). We predicted that honey bees kept in a landscape with a low amount of cultivation (i.e., corn and soybean production) would collect a greater quantity and diversity of pollen than those in a landscape with a high amount of cultivation. This prediction is based on an assumption that honey bees benefit and use forage available in noncropped features of the Iowa landscape (woodland, old fields and pastures, and semiurban to urban areas).

We also sought to understand the value of the most commonly collected pollen in this landscape, by focusing on the capacity of the pollen diet to support honey bee immune health. In addition to declining forage, pathogens, especially viruses, are considered a significant source of mortality for honey bees (Grozinger and Flenniken 2019). Augmenting the amount and type of pollen can improve honey bee immunity to pathogens (Parrinello et al. 2011, Foley et al. 2012, Di Pasquale et al. 2013). When provided a diet composed of pollen from diverse plants or from a single plant that produces high-quality pollen, honey bee survival was improved when infected with *Nosema* parasite and lethal viruses (Di Pasquale et al. 2013, Dolezal et al. 2019b). However, a diet of pollen from a single plant species of low-nutritional quality did not rescue bees from *Nosema* infection (Di Pasquale et al. 2013). Monofloral pollen of low quality reduced honey bee morality when infected with virus compared to bees denied pollen, but this mortality was higher than honey bees fed a ployfloral mixture of pollen (Dolezal et al. 2019b). However, the plants that were the source of pollen used in these studies are not relevant to Midwestern agricultural system. We determined the most commonly collected pollen by honey bees in central Iowa. This information was used to determine which plant species to include in an assessment of the value of pollen collected in central Iowa for protection from viral infection. We predicted that the differences in pollen diet found in central Iowa would affect honey bee resistance to virus infection.

## Materials and Methods

### Measuring the Impact of Land Use on the Diversity and Abundance of Pollen Collected by Honey Bees

#### Study sites

We summarize pollen foraging data from honey bee colonies that were part of a larger study exploring the impact of crop production on honey bee health (Dolezal et al. 2019a). This study demonstrated that landscapes surrounding apiaries of four colonies affected components of honey bee health (colony weight, adult and pupa populations, lipid concentration of individual nurse honey bees). Below we briefly summarize how the locations were selected.

**Fig. 1.**
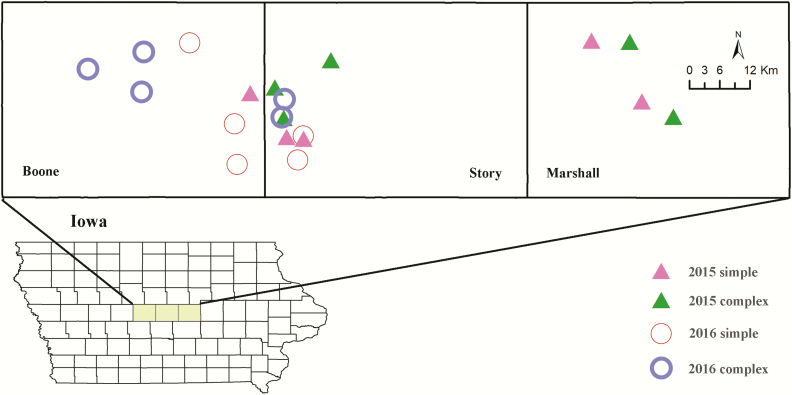
Location of apiaries within three counties of central Iowa, during 2015 and 2016. Apiaries were placed adjacent to soybean fields that were surrounded by landscapes that fitted two landscape categories (high vs low cultivation).

The colonies deployed in this study were kept in a three-county region of central Iowa, United States. To control for variation immediately adjacent to our honey bee colonies, we placed apiaries next to commercial soybean fields. In 2015, we selected 10 soybean fields in Boone, Marshall, and Story counties. Because soybeans are rotated yearly in central Iowa, locations changed between 2015 and 2016, resulting in a different set of 10 soybean fields in Boone and Story counties in 2016 (Fig. 1). To test our prediction that land use around a honey bee colony affected pollen foraging, we looked for soybean fields that were surrounded by a landscape that fell within two categories: low and high cultivation. We defined cultivation as the amount of corn and soybean grown within 1.6-km radius of the field edge where the honey bee colonies would be located. The percentage of these two crops that occupied the buffers around the colonies was calculated based on the amount of other land uses considered ‘noncrop’ (i.e., woodland, urban, pasture, and prairie). The amount of each land use type for a location was measured with ArcMap (Esri, Redlands, CA) from data collected by USDA-NASS (https://nassgeodata.gmu.edu/CropScape/). Details regarding the classification of noncrop area and specific details for each location can be found in the larger study (Dolezal et al. 2019a).

The average amount of noncrop habitat around fields within each category varied by year. For fields considered in the high cultivation category, the average amount of noncrop habitats was 18.4% and 13.6% in 2015 and 2016, respectively (Table 1). For fields in the low cultivation category, the average amount of noncrop habitat was 54.2% and 69.2% in 2015 and 2016, respectively. Previous studies have demonstrated that this low versus high cultivation classification scheme resulted in different communities of insects within a focal soybean field as well as differing nectar dynamics for honey bees (Gardiner et al. 2009, Bennett and Isaacs 2014, Dolezal et al. 2019a). By using these two extreme categories, we predicted that land use differences would provide significantly different amounts and diversity of flowering resources for our apiaries.

**Table 1.** High versus low cultivation landscapes assessed by proportion of area of crop (i.e., corn and soybean) and noncrop area in the landscape surrounding apiaries within 1.6-km radius

Landscape category	Mean ± SE % of noncrop habitat (range of noncrop habitat)	
	2015	2016
High cultivation	18.40 ± 3.78 (7–26)	13.60 ± 2.64 (7–21)
Low cultivation	54.20 ± 4.72 (44–70)	69.20 ± 8.67 (48–88)

In each year, five fields were selected to represent each landscape category.

An apiary of four colonies was placed at the field edge of each soybean field (5 per landscape category per year) that was managed using conventional practices with regards to pesticides (fungicides and herbicides), fertilizer, and tillage use. Because honey bees have been estimated to mainly forage (around 90% visitation) for pollen within a 1.6-km radius of their colonies in agricultural landscapes (Couvillon et al. 2014, Danner et al. 2014), we selected fields such that any two apiaries were at least 3.2 km apart from each other. In this way, we attempted to limit overlapping honey bee foraging ranges between fields of different landscapes so that the pollen collected from each apiary could be considered independent. This distance resulted in average colony weight varying significantly between apiaries kept at soybean fields in low versus high cultivation landscapes (Dolezal et al. 2019a).

#### Honey bee apiaries

An apiary of four colonies housed in Langstroth hives was placed together on a wooden pallet at the field edge of each soybean field. These colonies were part of the larger experiment and a more detailed response of colonies to the two landscape categories is described in terms of differences in colony weight, brood amount, adult bee population (Dolezal et al. 2019a). To reduce the potential negative effect of pollen traps on colony health, only one of the four colonies at each field was used to measure pollen collection, but the management was the same for all colonies within an apiary.

Apiaries were first established at an Iowa State University (ISU) farm and then distributed to our study fields on 10 June 2015 and 23 May 2016. In 2015, the colonies were started from 0.9 kg packages of bees (about 7,000 bees); while in 2016, colonies were started from nucleus colonies consisting of approximately three frames of worker bees (similar amount to 2015). To reduce variation due to genetic lineage, queens used in all colonies in both years were *Apis mellifera ligustica*. Each colony was inspected once every 14 d as part of a standard protocol to measure colony health, including checking for the presence of the queen. If we did not observe the queen or sign of her activities (i.e., egg or young larvae), the colony was re-queened within 1–2 d with a queen from the same commercial source from which colonies were derived. Colonies were not fed supplementary feed throughout the experiment. Other apiary management details were demonstrated in another journal publication (Dolezal et al. 2019a).

#### Pollen collection

One colony in each apiary was randomly selected to receive a pollen trap attached to the colony entrance (Brushy Mountain Bee Supply, Wilsonville, OR). Foraging honey bees must enter the trap to return to the colony entrance, and a plastic plate with many star-shaped holes in the trap pulls pollen pellets from corbiculae of individual bees. Dislodged pollen pellets fall into a basket under the trap. When not in use, the plastic plate was removed, allowing foragers to return undisturbed into the colony.

In total, 10 pollen collections were taken from July to September in 2015, and 13 from June to September in 2016. Those pollen collections resulted into 100 pollen samples in 2015 and 130 samples in 2016. Pollen traps were opened for 24 h on each collection without rain. After the nonpollen debris was removed, pollen samples were weighed and stored at −20°C for later taxonomic identification.

#### Pollen identification

We used a compound light microscope to view morphological features of the pollen grains to identify from which plant species they were collected. A 2-g subsample of pollen collected at each field and date was first sorted according to color. Pollen of different colors was weighed and dissolved in Caberla’s solution using fuschin dye and then mounted onto glass slides. To identify pollen to the lowest taxonomic level, pollen from the traps was compared to pollen extracted from flowers obtained from the study areas during the period when pollen traps were open. Pollen types that were not identified based on this reference collection were recorded as unknown and given a separate morpho-species designation. Pollen diversity was assessed by species richness (number of plant taxa represented by the pollen) and the species richness and evenness of plant taxa represented in a collection was assessed using Shannon’s diversity index.

### Measuring the Effect of Variation in Pollen Diet on Honey Bee Immune Health

To test if pollen from the most commonly collected species within the central Iowa landscape affected honey bees’ resistance to viral infection, we conducted a laboratory-based experiment on the campus of ISU in 2017. There were two experimental factors: virus infection (two levels; present or absent) and diet source (four levels; described below), accounting for eight treatments, with 24 replicates per treatment for a total of 192 experimental units. Each experimental unit consisted of a cage containing 30 newly emerged honey bees.

The no-pollen diet served as a negative control and chestnut (*Castanea* spp., purchased from Pollenergie, Saint-Hilaire-de-Lusignan, France) as a positive control. Chestnut was selected as it has high levels of protein and antioxidants, and in a similar assay was observed to rescue honey bees from a lethal dose of the microsporidian pathogen *Nosema ceranae* (Di Pasquale et al. 2013) and a mixture of viruses (Dolezal et al. 2019b). The other two pollen diets were based on the most commonly collected pollen from our field experiment: clover pollen (*Trifolium* spp.) and a 50%:50% mixture of clover and partridge pea (*Chamaecrista fasciculata*). Clover pollen consisted of approximately 50% red clover (*T. pratense*) and 50% white clover (*T. repens*) pollen. The pollen diets were fed to bees by mixing three portions of each pollen group with one portion of 50% sucrose water free of virus.

The virus inoculum was produced according to methods used by Carrillo-Tripp et al. (2016) and Dolezal et al. (2019b). Five common virus types were screened in our inoculum, including acute bee paralysis virus (ABPV), black queen cell virus (BQCV), deformed wing virus (DWV), Israeli acute paralysis virus (IAPV), Kashmir bee virus (KBV), and sacbrood virus (SBV). Of these, only IAPV, BQCV, and SBV were detected as follows: 85% IAPV, 14% SBV, and 1% BQCV. Primers used for identification and quantification of virus compositions were the same as those used in the two previous studies. Virus inoculum in phosphate-buffered saline (PBS) was diluted with 30% sucrose solution by 1:750 ratio prior to the feeding. Caged bees were infected by consuming the 600 µl sucrose solution containing virus inoculum. Our no-virus infection controls were fed a sterile sucrose solution identical to that used in the treatment except with 0.8 µl sterile PBS containing virus particles. Previous work has shown that, using these procedures, IAPV is the primary replicating virus, including when SBV is present in even higher quantities, and is the cause of honey bee mortality (Carrillo-Tripp et al. 2016, Dolezal et al. 2019b).

Honey bees used in this experiment were collected from brood frames identified with emerging bees from five colonies at the ISU apiary. All adult bees were removed from these frames before they were brought to a laboratory on the campus of ISU. These capped brood frames were kept in wooden boxes within a rearing room (33°C and 80% RH). After 24 h, newly emerged bees were collected, mixed, and randomly assigned to 192 acrylic cages (eight treatments comprised of 24 cages per treatment) with 30 bees per cage (10.6 × 10.16 × 7.62 cm).

The four pollen diets were randomly assigned to each cage with newly emerged honey bees, with 2.2 g pollen placed at the bottom of each cage. The pollen diets were renewed every 24 h. Half of all replicates (96 cages) were randomly assigned exposure to the virus mixture. A volume of 600 µl diluted virus solution was provided to honey at a plastic bowl on bottom of the cage bees immediately after feeding pollen diets for the first time. The remaining 96 cages were provided with 30% sucrose solution without virus. Once the virus solution was consumed, all bees were fed ad libitum with a virus-free 50% sucrose solution through 15-ml plastic tubes on the top of the cage during the rest of the experimental period. Dead bees were removed every 24 h and the amount recorded to calculate % mortality.

#### Statistical analysis

To determine the effect of varying landscapes on the abundance and diversity of pollen forage to honey bees, we used a repeated measure design by monitoring pollen collection throughout the growing season. Linear mixed models were used to conduct an analysis of variance (PROC MIXED) on pollen amount and diversity using SAS 9.3 software (SAS Institute, Cary, NC). Pollen amount (g) and diversity (species richness and Shannon diversity) was the dependent variable, with landscape as the independent variable, date as the repeated variable, and field as a random effect in the analysis. We used an AR1 (autoregressive) structure (PROC MIXED) for the correlation of amount of pollen or diversity collected among different dates to obtain the lowest AIC and AICC value in the above model. Honey bee colony population grows over time and, to reduce the variation of colony size on foraging behavior, we standardized the amount of pollen collected by colony weight, which included the mass of adult bees, brood, honey, and pollen. To improve the normality of the pollen abundance data, the amount of pollen was transformed (base-10 log) prior to analysis when necessary. Due to the difference between colony arrival dates to the fields and starting colony size, the amount of pollen collected was analyzed separately for both years. Pollen amount and diversity from the two landscape categories at each date was also compared using least square means under the condition of a linear mixed effects model.

To describe the patterns of pollen collected over time, the average amount of pollen of different months (normalized by average colony mass of each month) was compared using analysis of variance (PROC MIXED) and Tukey–Kramer HSD multiple comparisons. To demonstrate how major pollen types changed over time, we organized the source plants of pollen collection into three groups: clover, partridge pea, and trace pollen. Because white clover and red clover are from the same genus and have similar blooming periods in central Iowa, we included them into one group. Pollen from plants that represented <5% of all pollen collected was grouped as trace pollen.

The cage experiment was conducted as a fully crossed, completely randomized design, with every combination of virus and diet treatment represented in the analysis. To test the efficacy of the virus treatment, mortality of virus-treated honey bees was compared with that of untreated honey bees using a Welch’s two sample *t*-test (PROC TTEST). To determine if different pollen diets affected honey bee survival when challenged by the virus, the % mortality of honey bees fed by different diets was analyzed within virus-treated bees by analysis of variance (PROC MIXED).

## Results

### Pollen Abundance in Apiaries Within Differing Landscapes

**Fig. 2.**
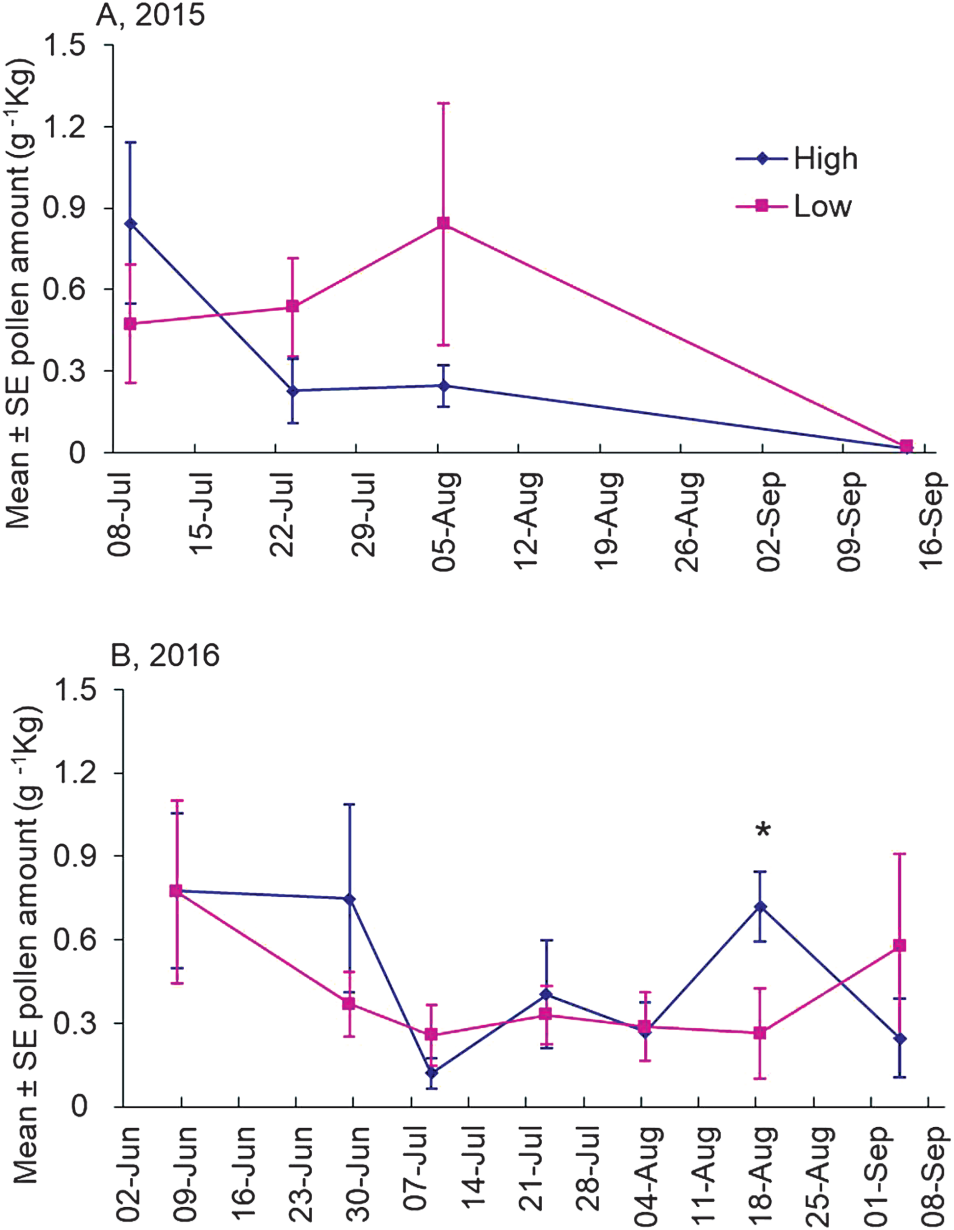
The abundance of pollen collected by honey bees in high and low cultivation landscapes of central Iowa during 2015 (A) and 2016 (B). The weight (g) of pollen collected was normalized by net colony weight (kg), resulting in the use of g^−1^kg along the y-axis. Pollen abundance did not statistically differ between the two landscape categories (Table 2). Note this analysis was conducted on a subset of dates that included only days when colony weight was measured (excluding the pollen data without available corresponding colony weight for normalization). * Indicates a statistically significant difference on a single date by least square means under the mixed effects model (*P* < 0.05).

The amount of pollen collected by honey bees throughout the sampling period did not differ between colonies located in either the low or high cultivation categories in 2015 (*F* = 0.13; df = 1, 10.3; *P* = 0.728; Table 2; Fig. 2A) or 2016 (*F* = 0.64; df = 1, 15.10; *P* = 0.437; Table 2; Fig. 2B). On one specific date, 18 August 2016, the amount of pollen collected in the high cultivation landscapes was significantly greater than that from the low cultivation landscapes (Fig. 2B, *t* = −2.18; df = 49.7; *P* = 0.034). Otherwise, there were no significant differences in the amount of pollen collected between the two landscape categories on any other date in both 2015 and 2016.

**Table 2.** Repeated measure ANOVA for pollen abundance and diversity in 2015 and 2016 using linear mixed effects model

Pollen	Source of variance	df	*F*-value	*P-*value
Abundance	2015			
	Landscape	1, 10.3	0.13	0.728
	Date	3, 21.6	19.26	<0.000
	Landscape × date^*a*^	3, 21.6	2.36	0.099
	2016			
	Landscape	1, 15.1	0.64	0.437
	Date	6, 42.2	0.79	0.584
	Landscape × date	6, 42.2	0.92	0.488
Taxonomic richness	2015			
	Landscape	1, 17.4	0.20	0.659
	Date	9, 65.8	6.28	<0.000
	Landscape × date	9, 65.8	0.38	0.939
	2016			
	Landscape	1, 18.3	0.41	0.528
	Date	12, 82.6	1.33	0.220
	Landscape × date	12, 82.6	0.37	0.971
Shannon diversity	2015			
	Landscape	1, 22.2	0.01	0.929
	Date	9, 65.4	2.71	0.010
	Landscape × date	9, 65.4	0.36	0.948
	2016			
	Landscape	1, 22.7	1.31	0.265
	Date	12, 86.7	1.95	0.039
	Landscape × date	12, 86.7	0.64	0.800

^*a*^Interaction between landscape and date.

### Diversity of Plant Species Used for Pollen Forage

In 2015, both landscapes categories had 25 plant taxa found in our collection (Table 3; Supp Tables 1 and 3 [online only]). Among plant taxa identified in our pollen, 11 taxa were shared between the two landscape categories; among pollen from unidentified plant taxa, only four were shared between the two landscape categories (Supp Table 3 [online only]). Six native plant taxa were used by honey bees with four of these shared between two landscape categories. *Ratibida pinnata* (Vent.) Barnhart (Asterales: Asteraceae) and *Phlox paniculata* L. (Ericales: Polemoniaceae) were collected only by colonies in the low cultivation categories (Table 3).

**Table 3.** Taxa of plants identified in the pollen collected by honey bees during 2015

Plant taxa^*a*^	% of each pollen type (mean ± SE) by weight	
	High cultivation	Low cultivation
*Triflolium repens*	42.13 ± 10.66	34.38 ± 15.84
*Trifolium pretense*	24.66 ± 10.08	32.39 ± 8.64
*Chamaecrista fasciculate* ^*b*^	24.37 ± 8.44	26.77 ± 12.11
*Solidago* spp.^*b*^	3.10 ± 1.69	2.10 ± 0.85
*Circium vulgare*	2.15 ± 1.43	0.87 ± 0.38
*Lotus corniculatus*	0.90 ± 0.86	0.13 ± 0.13
*Helianthus, Heliopsis, Silphium* spp.^*b*^	0.63 ± 0.41	0.27 ± 0.19
*Sambucus canadensis*	0.59 ± 0.59	0
*Ambrosia* spp.	0.30 ± 0.10	0.64 ± 0.39
*Melilotus* spp.	0.14 ± 0.14	0.81 ± 0.81
*Dalea purpurea* ^*b*^	0.13 ± 0.11	0.05 ± 0.05
*Zea mays*	0.06 ± 0.06	0.18 ± 0.18
*Saponaria officinalis*	0	0.29 ± 0.29
*Ratibida pinnata* ^*b,c*^	0	0
*Phlox paniculat* ^*b*^	0	0.24 ± 0.24
Unknown taxa^*a*^	0.82 ± 0.43	0.87 ± 0.44

^*a*^In total, 18 unrecognized pollen types were combined into ‘unknown taxa’, but % of each unrecognized pollen type was informed in Supp Table 1 (online only). Pollen types were arranged in the order of high to low percentage of simple landscape.

^*b*^Native plants.

^*c*^Pollen was less than < 0.01%.

In 2015 clover was the most abundant pollen by mass, while partridge pea was the other abundant pollen collected by honey bees (Table 3; Supp Figs. 1 and 2 [online only]). Over 90% of the total pollen brought back to the colonies throughout the entire experimental period was comprised of clover (*Trifolium* spp.) and partridge pea (*Chimaechrista fasciculate*) in both landscape categories (Table 3). Among 100 pollen samples collected on different days during 2015, 50 ones were composed of >90% clover.

In 2016 a total of 51 and 54 plant taxa found in bee-collected pollen were from high and low cultivation landscape, separately (Table 4; Supp Tables 2 and 3 [online only]). In total, 21 plant taxa identified in pollen were shared between two landscape categories, so did 20 plant taxa unidentified in pollen (Supp Table 3 [online only]). Ten native plant taxa were used by honey bees with eight of these shared between two landscape categories (Table 4; Supp Table 3 [online only]). The native plants *Zizia aurea* L. (Apiales: Apiaceae) and *R. pinnata* were collected only by colonies in the high cultivation landscape (Table 4). Clover (*Trifolium* spp.) and partridge pea accounted for over 73% of the total pollen collected in the entire experimental period identified in both landscapes during 2016 (Table 4). A number of 39 out 130 pollen samples were composed of >90% clover.

**Table 4.** Taxa of plants identified in the pollen collected by honey bees during 2016

Plant taxa^*a*^	% of each pollen type (mean ± SE) by weight	
	Simple	Complex
*Trifolium pretense*	34.38 ± 7.33	39.42 ± 9.70
*Triflolium repens*	30.72 ± 4.45	27.51 ± 7.75
*Chamaecrista fasciculate* ^*b*^	10.83 ± 6.60	5.71 ± 5.24
*Lotus corniculatus*	4.69 ± 4.23	1.59 ± 0.96
*Melilotus* spp.	3.88 ± 1.04	1.94 ± 0.57
*Ambrosia* spp.	2.09 ± 0.54	3.71 ± 2.07
*Circium vulgare*	1.76 ± 1.63	0.83 ± 0.44
*Dalea purpurea* ^*b*^	1.42 ± 1.34	0.01 ± 0.01
*Zea mays*	1.21 ± 0.71	1.93 ± 1.40
*Iris versicolor* ^*b*^	0.19 ± 0.09	1.90 ± 1.87
*Taraxacum officinale*	0.17 ± 0.13	0.23 ± 0.15
*Pastinaca sativa*	0.17 ± 0.11	0.16 ± 0.08
*Saponaria officinalis*	0.15 ± 0.11	3.95 ± 3.63
*Tilia americana* ^*b*^	0.13 ± 0.09	3.63 ± 3.63
*Asparagus officinalis*	0.12 ± 0.12	0
*Solidago* spp.^*b*^	0.08 ± 0.04	0.49 ± 0.45
*Daucus carota*	0.05 ± 0.03	0.42 ± 0.32
*Phlox paniculata* ^*b*^	0.04 ± 0.04	0.17 ± 0.15
*Helianthus*, *Heliopsis*, and *Silphium* spp.^*b*^	0.04 ± 0.03	0.15 ± 0.11
*Cichorium intybus*	0.03 ± 0.03	0
*Sambucus canadensis*	0.03 ± 0.02	0.02 ± 0.02
*Rudbeckia hirta*	0.02 ± 0.01	0.02 ± 0.02
*Zizia aurea* ^*b*^	0.01 ± 0.01	0
*Hemerocallis fulva*	0.01 ± 0.01	0.03 ± 0.03
*Ratibida pinnata* ^*b*,*c*^	0	0
*Verbena stricta* ^*b*^	0	0.16 ± 0.14
Unknown taxa^*a*^	7.78 ± 3.95	8.27 ± 1.58

^*a*^In total, 38 unrecognized taxa combined into ‘unknown taxa’, but % of each unrecognized taxa was informed in Supp Table 2 (online only).

^*b*^Native plants.

^*c*^Pollen was less than <0.01%.

**Fig. 3.**
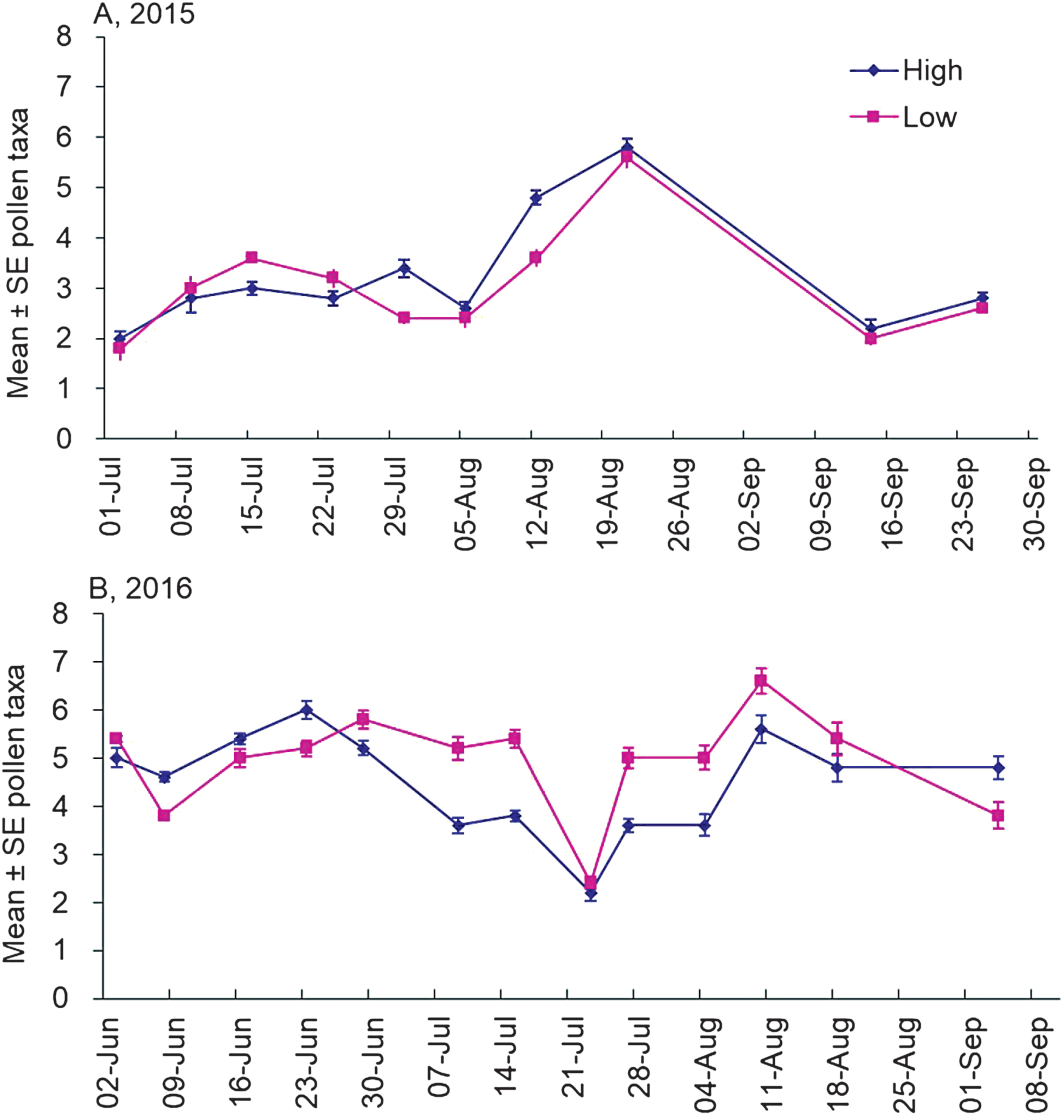
Taxa richness of pollen returned to honey bee colonies in two different landscapes of central Iowa during 2015 (A) and 2016 (B). No significant difference was observed between the two landscape categories (Table 2).

**Fig. 4.**
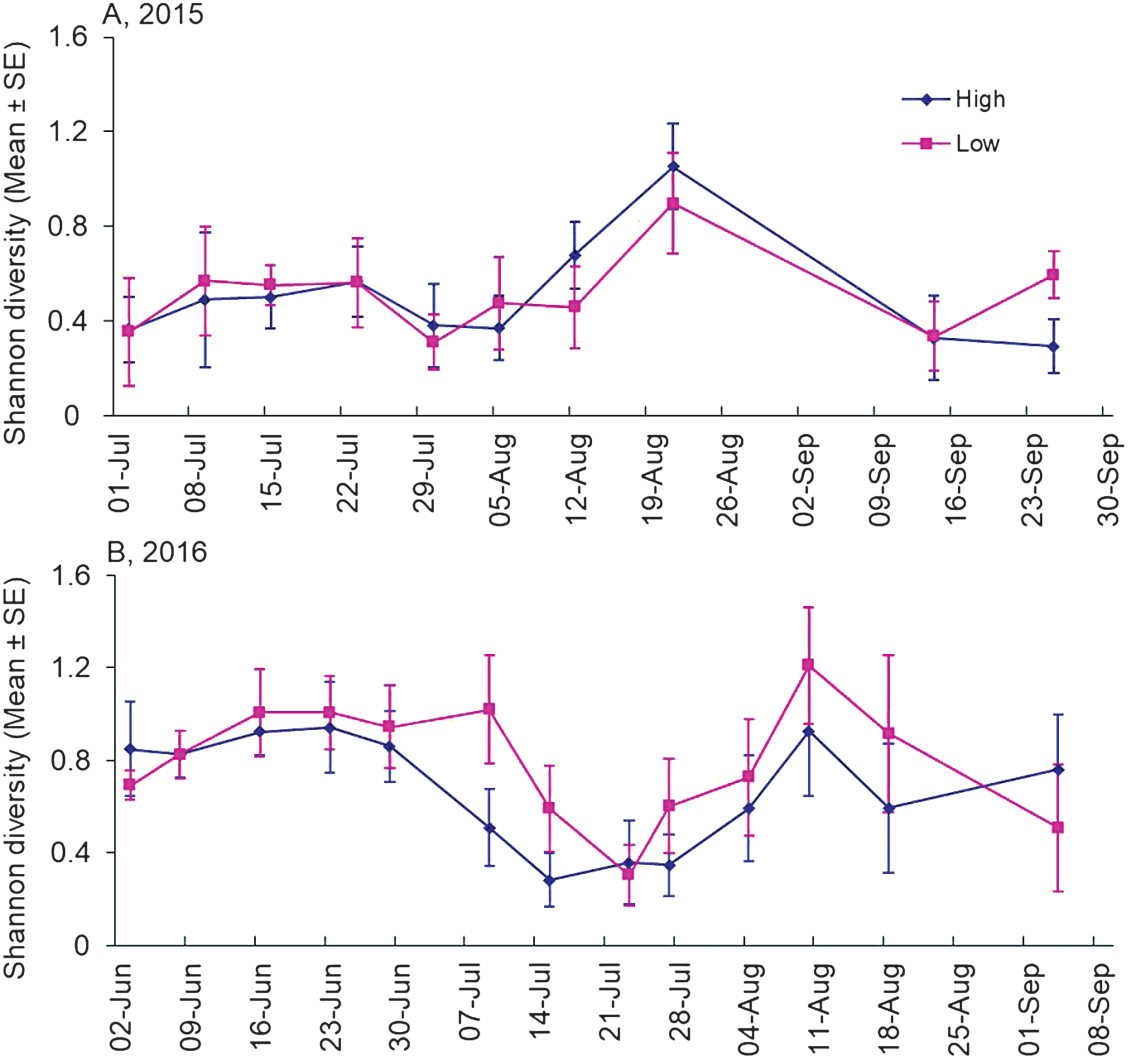
The diversity of pollen returned to honey bee colonies in two different landscapes of central Iowa as estimated with the Shannon diversity index during 2015 (A) and 2016 (B). No significant difference was found between the two landscape categories (Table 2).

Taxa richness and Shannon diversity of pollen brought to colonies did not significantly differ between landscape categories in both years (*P* > 0.05) (Table 2). Across individual dates in both years, we did not observe any difference in the richness and Shannon diversity of pollen between low versus high cultivation landscapes (*P* > 0.05 for all dates) (Figs. 3 and 4). Taxa richness was generally in the range of 2–6 taxa per 24 h sampling period in 2015 and 2016 (Fig. 3). Shannon diversity index had similar patterns as richness (Fig. 4).

In summary, the diversity of plants used by bees in the two landscape categories was very similar. In 2015, there were no differences in the plants used and in 2016, only two more taxa were found in the pollen of colonies kept in the low cultivation landscape. Therefore, we conclude that locating bee colonies in low cultivation landscapes did not increase the number of plants taxa used by honey bees for pollen forage. And a larger portion of plant taxa foraged by honey bees were shared between the two landscapes. Among the plant taxa that were identified in the pollen, all the native plants can be found in grasslands and prairies (Tables 3 and 4; Supp Table 3 [online only]), except *Tilia americana* which is a woodland species. The non-native plant taxa were most likely found in agricultural components of landscape (Tables 3 and 4; Supp Table 3 [online only]).

Despite being next to soybean fields, we did not observe any soybean pollen in the pollen traps during both years. However, other legumes, clover and partridge pea, were the most commonly collected pollen in both years (Tables 3 and 4). Corn was rarely a source of pollen (<1% in 2015 and <2% in 2016) though corn was very abundant.

### Phenology of Pollen Forage

**Fig. 5.**
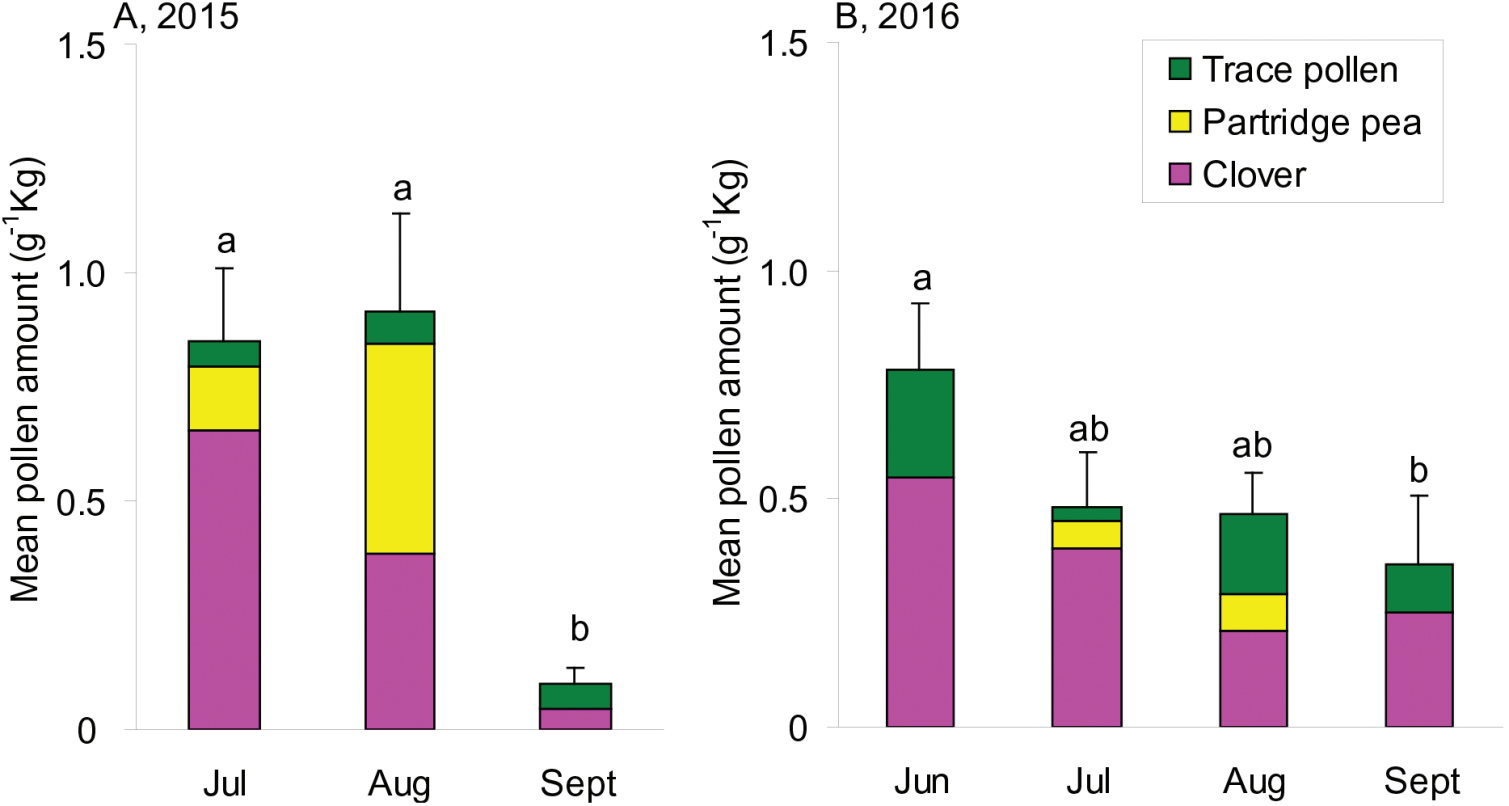
The community composition of pollen collected by honey bees in two categories of central Iowa landscapes by month during 2015 (A) and 2016 (B). Columns represent the total amount of pollen collected and colors within a column indicated plant species. Plants were included in the group of ‘trace pollen’ if they contributed less than 5% of the total by weight. See Tables 3 and 4 and Supp Tables 1 and 2 (online only) for a list of species represented in the pollen collection. Error bars represent standard errors of averaged total pollen. Different letters above error bars indicated the significant differences of averaged total pollen among months according to ANOVA results followed by Tukey–Kramer HSD multiple comparisons.

Although we did not detect a difference in the amount of pollen collected by honey bees kept in the low versus high cultivation categories, we did observe differences in the amount of pollen collected by sampling date (Table 2). When the amount of pollen was organized into three general groups (i.e., clover, partridge pea, and trace pollen) and binned by month, we observed a remarkable decline in the amount of pollen returned to the colonies (Fig. 5). In both 2015 and 2016, honey bees collected the least amount of pollen by weight during September (Fig. 5). The amount of pollen collected in September were only 12% and 46% of its peak weight in 2015 and 2016, separately.

### Variation in Pollen Diets Affects Honey Bee Immune Health

**Fig. 6.**
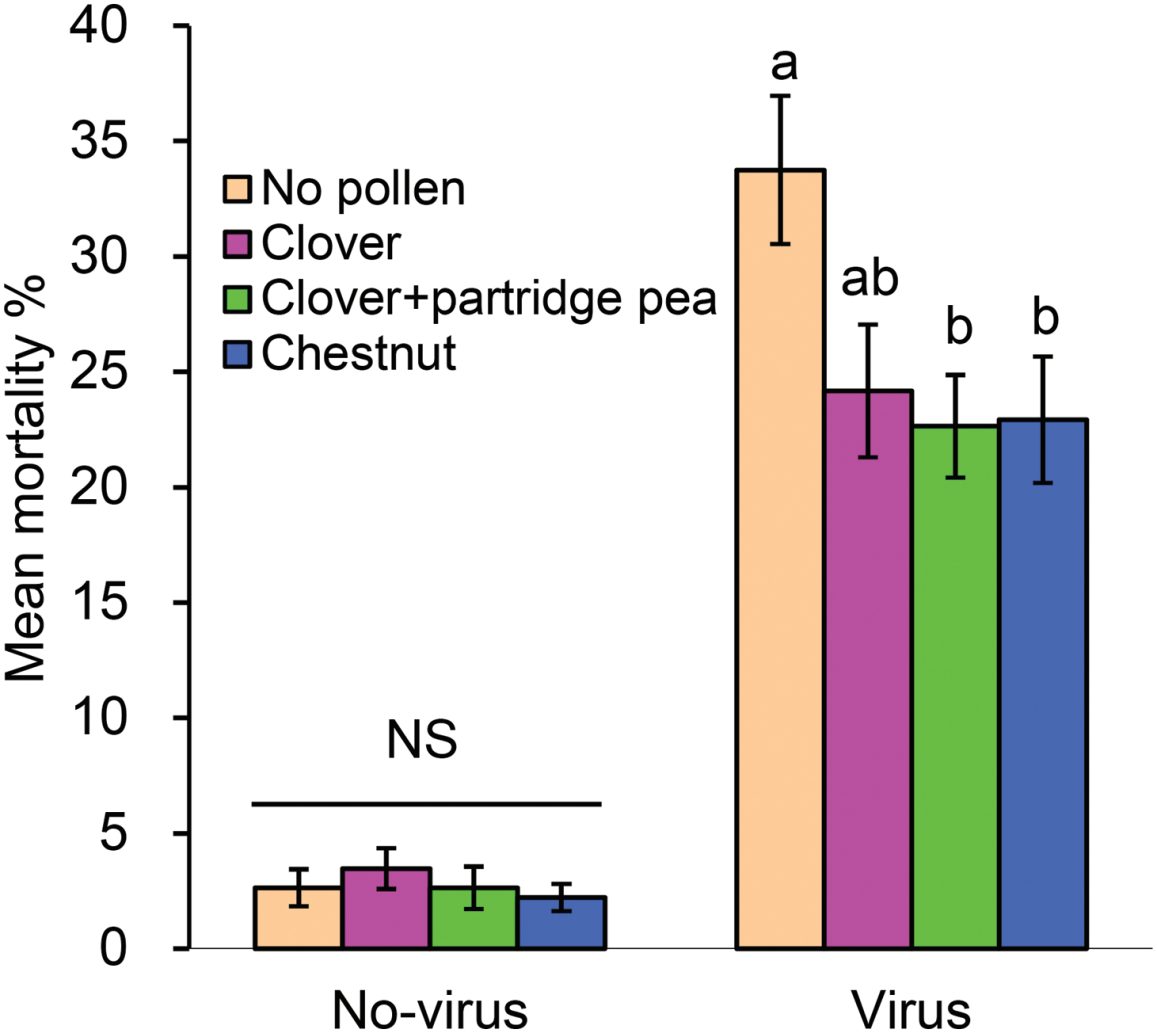
Mortality of caged honey bees either uninfected or infected with a mixture of viruses. Each group of bees were provided four differing pollen diets and ad libitum sucrose solution. Mortality is reported as the percent of individual bees that died after 72 h within each treatment. The mortality of bees infected with virus was significantly higher than uninfected bees (*t* = 15.39; df = 109.48; *P* < 0.0001). Different letters above the standard error bars indicate significant differences among the diets for virus-infected bees (*F* = 3.62; df = 3, 95; *P* = 0.016, multiple comparison by Tukey–Kramer HSD). Mortality of honey bees uninfected with virus did not significantly (NS) differ by pollen diet (*F* = 3.62; df = 3, 95; *P* = 0.016).

Using a method that has been shown to reveal variation in honey bee mortality to viral infection based on diets (Carrillo-Tripp et al. 2016, Dolezal et al. 2019b), we found honey bees receiving the virus treatment suffered significantly higher mortality than those untreated (*t* = 15.39; df = 109.48; *P* < 0.0001) (Fig. 6). In the absence of a viral infection, there was no significant difference in percent mortality among the pollen diets, including the no-pollen diet (*F* = 0.42; df = 3, 95; *P* = 0.740, multiple comparison by Tukey–Kramer HSD). However, for honey bees receiving the virus treatment, we observed a significant difference in mortality between infected honey bees provided with the various pollen diets (*F* = 3.62; df = 3, 95; *P* = 0.016). Our positive control (i.e., *Castanea* pollen) confirmed that our assay could detect improvements in honey bee survival consistent with previously published studies (Di Pasquale et al. 2013, Dolezal et al. 2019b). Clover (*Trifolium* spp.) pollen alone did not significantly reduce honey bee mortality compared to the no-pollen diet; however, the mixture of clover and partridge pea pollen significantly reduced mortality by 10% compared to the no-pollen diet (*F* = 3.62; df = 3, 95; *P* = 0.016, multiple comparison by Tukey–Kramer HSD).

## Discussion

### Equal Abundance and Diversity of Pollen Collection Between Two Landscape Categories

Our initial prediction that landscape variation around honey bee colonies kept adjacent to soybean fields would affect the amount and diversity of pollen collected by foraging honey bees was not confirmed. We predicted that both the abundance and diversity of pollen would be greater in colonies kept in the landscapes with low cultivation, as these would have a greater diversity of plants beyond corn and soybean. As anticipated, honey bees did not use either corn or soybean as a significant source of pollen (Tables 2 and 3), indicating that these crops did not directly influence the pollen foraging behavior of honey bees. Contrary to our prediction, the abundance and diversity of pollen collected by honey bees did not vary between low and high cultivation landscapes even though fields within the low cultivation landscape contained more noncrop habitats than fields within the high cultivation landscapes.

These results were similar to previous studies in which the amount and diversity of pollen collected by honey bees was measured across colonies kept in multiple locations that varied in the diversity of land use surrounding honey bee colonies (Smart et al. 2016a,b; Danner et al. 2017). There could be several explanations for why variation in land use did not affect the amount and diversity of pollen collected by honey bees in these studies. The simplest explanation may be that the occurrence of plants that represent the most commonly collected pollen (clover species, *Trifollium repens* and *T. pretense*, and partridge pea, *C. fasciculata*) did not differ between the two landscape categories in our study. These two plant taxa are not intentionally planted in either landscape category in our study. The first most commonly collected pollen (clover species) came from plant species (clover) that are well-known sources of forage for honey bees (Sponsler and Johnson 2015). Although these clover species are not native to North America, it is common throughout the Midwest, in part because was intentionally added to pastures for livestock production. However, land committed to pasture currently makes up a small portion of the central Iowa landscape (Dolezal et al. 2019a) and clover is widely distribute in roadside, field margins, and lawns as a weedy species (Turkington and Burdon 1983, Sponsler and Johnson 2015). These locations were components of both the low and high cultivation landscapes in Iowa where flowering clovers were found (G.Z., personal observations). Clovers have a long blooming period from summer to early fall (Turkington and Burdon 1983, Larson et al. 2014) that could be a source of honey bee forage. By the end of August, clover ceased to bloom and honey bee colonies begin to lose weight comprised mostly of honey (Dolezal et al. 2019a). Interestingly, the second most commonly collected pollen came from partridge pea (*C. fasciculata*) after clover bloom. It is a native North American annual plant whose native habitat (prairie) has been reduced to less than 0.01% of its original range. Other land uses such as roadside, river banks, and conservation land that are component of both landscape category were potential habitats for partridge pea used for versatile purposes such as cover crops for erosion control and improving soil fertility, forage for wild life, and recreation (Hardin et al. 1972, Kauffeld 1980, Mannouris and Byers 2013, Houck and Row 2019). This is remarkable as honey bees are not native to Iowa but seem to prefer these rare sources of forage over more abundant sources of pollen, such as corn and soybean. Habitat that could potentially contain both clover and partridge pea represent a large amount of the area in both landscape categories, so estimating the floral population of these two plants is beyond the resources available to us at the time of this study. Clover pollen was found in all pollen traps during both years and partridge pea found in the majority of traps (at eight fields during 2015 and seven fields during 2016), suggesting that clover and partridge pea plants may be ubiquitous in central Iowa.

### Phenology of Pollen Availability

Because honey bees forage throughout the growing season, there is a need to explore the response to landscapes over a phenological period that extends beyond the flowering period of the dominant crop(s) or noncrop sources of forage. We observed variation in pollen abundance over the season. As the amount of clover collected in colonies declined in August, partridge pea became more of the total pollen brought to colonies (Fig. 5; Supp Figs. 1 and 2 [online only]). The reduction in clover pollen occurred during as clover ceased blooming (Dolezal et al. 2019a), and likely not due to a greater attractiveness of partridge pea. Honey bees may have turned to partridge pea for enough forage leading to similar overall amount of pollen from both plants collected within the two landscape categories. Previous studies in Europe have identified August as a period of pollen dearth in temperate regions (Garbuzov et al. 2015, Requier et al. 2015, Danner et al. 2017), and this food shortage is considered to contribute to colony losses (Requier et al. 2017). Partridge pea blooming in August within central Iowa is an alternative source of pollen not readily available during the same time period in Europe.

By September, we observed a significant reduction in pollen brought back to the colonies. In September of both years, clover was still part of this collection, partridge pea was not found, and a mixture of other plants became a source of pollen. Identifying a period of pollen shortage provides valuable information for aiding bee nutritional health by indicating when there is a need for alternative forage. Lower pollen availability in September may be critical for honey bees preparing for overwintering (Fig. 5). Some native *Solidago* spp. and *Helianthus* spp. were sources of pollen for bees later in the growing season (Tables 2 and 3). These species typically bloom during August and September and could help counter a shortage of pollen during September if seeded or planted near apiaries (Ginsberg 1983, Smart et al. 2016b, Wood et al. 2018). Future studies could focus on determining the value of plants that bloom in the later part of the growing season (e.g., August, September) when bee colonies rear new bees for overwinter that is critical for general colony health and overwinter survivorship.

### Enhanced Resistance to Viral Infection: A Potential Benefit From a Diet of Two Pollen Sources

Regarding our second prediction that varying pollen diet affect honey bee health, we selected pollens observed in our field study to determine their contribution to an aspect of colony health, i.e., resistance to virus infection. We compared a pollen diet composed of a mixture of pollen from two clover species (*Trifolium* spp.) to a mix of clover and partridge pea in proportions similar to what we observed in the field. Clover pollen alone did not significantly reduce honey bee mortality from a viral infection compared to no-pollen diet. When honey bees were provided pollen from both clover and partridge pea, mortality was significantly reduced compared to a no-pollen diet. These results suggested that partridge pea may be more than just an alternative source of pollen late in the season but also an improvement in the quality of the honey bee diet. Given that pathogens and forage availability are considered key stressors experienced by honey bee and multiple species of wild bees, these data were interesting as they suggested an explanation for why honey bees are using a native plant as a source of pollen. These data also suggested that by conserving habitat that contains this native plant (and others potential sources of pollen after clover ceases to bloom), honey bees may be relieved of these stressors. Honey bees collected pollen from several other plant species throughout the course of this study (i.e., trace pollens), though in much lower quantities than either clover or partridge pea. To what extent these trace pollens could sufficiently improve the survival of honey bee’s resistance to viral infection needs further exploration.

### Value of Legumes for Honey Bee Pollen Forage

Remarkably, several of the plants used by honey bees as a forage in central Iowa are legumes. Six legumes species were common sources of pollen regardless of where colonies were located, including non-native white clover (*Trifolium repens*), red clover (*Trifolium pratense*), sweet clover (*Melilotus* spp.), birdsfoot trefoil (*Lotus corniculatus* L. [Fabales: Fabaceae]), and native partridge pea (*C. fasciculata*) and purple prairie clover (*Dalea purpurea* Vent. [Fabales: Fabaceae]). Combined, these plants represented 93% or 81% of the total pollen collected by honey bees in 2 yr of our study (Tables 3 and 4; Supp Fig. 3 [online only]). Although colonies were placed adjacent to fields of soybean, also a legume, we did not observe soybean pollen in any of the colonies. Previous studies have also demonstrated that at least one of those legumes found in our study was a major source of pollen for honey bees in other states of the Midwestern United States, including Kansas (Rashad 1955), Minnesota (Smart et al. 2016b), North Dakota (Smart et al. 2016b), Indiana (Long and Krupke 2016), Ohio (Sponsler et al. 2017), Wisconsin (Severson 1978). Except for red clover, the other five legume species are also considered to be a significant source of nectar for the production of a honey crop (Sweet 1949).

These six legume species were also recommended by Decourtye et al. (2010), as opportunities for the enhancement of pollen forage in agricultural landscapes. If planted as bee forage, care must be taken because some non-native legumes, e.g., birdsfoot trefoil (Williams and Smith 2007, Gerla et al. 2012) and sweet clover (Cole 1991, Wolf et al. 2003, Conn et al. 2011), can invade and colonize native habitats (i.e., prairie). Less invasive legumes like white clover, red clover, and native legumes like purple prairie clover and partridge pea are more suitable choices for increasing source of pollen for honey bees in agricultural landscapes like Iowa. In addition, legumes used as cover crops can improve soil nutrition by fixing nitrogen via root symbiosis, thus contributing to stacked benefits for both agronomical and apicultural management.

In conclusion, we did not observe an effect of low versus high cultivation landscapes, surrounding colonies on the amount and diversity of pollen collected by honey bees. In general, honey bees in central Iowa were able to collect pollen even in landscapes dominated with corn and soybean production (i.e., high cultivation). Regardless of the varying surrounding landscapes, a few species of plants considered as attractive bee forage were consistently discovered by honey bees, primarily multiple species of legumes (mainly clover and partridge pea). This was true even for colonies located in fields in which 90% of the land used within a 1.6-km radius was corn and soybean. Although the diversity of our honey bees’ pollen diet was generally low, we determined that by feeding on a pollen diet consisting only of a two taxa of legumes, honey bees experienced reduced mortality from viral infections. This suggests that even small improvements in forage diversity has the potential for improving the health of honey bees.

If honey bees deliberately acquire pollen from different species to satisfy their nutritional needs (Hendriksma and Shafir 2016), this may have been challenging in the agricultural landscape of central Iowa especially early in the growing season when clover dominates the pollen brought back to colonies (Fig. 5; Supp Figs. 1 and 2 [online only]). Some native plants can be planted in agricultural land dominated by corn and soybean to increase forage diversity in early season. For example, sunflower (*Helianthus annuus* L. [Asterales: Asteraceae]) can have medication effect on bees, and could be an option for forage enhancement (Jonathan et al. 2018, LoCascio et al. 2019). Efforts to conserve beneficial insects have revealed that native plants commonly found in prairies, the dominant habitat in Iowa before European settlement, are highly attractive to native pollinators as well as managed honey bees (Tuell et al. 2008, Blaauw and Isaacs 2014). Many of the flowering forbs found in those prairies, such as native sunflower and goldenrod, may be a potential forage source for honey bees that surfer from lack of pollen availability in later season observed in this study. Dolezal et al. (2019a) demonstrated that prairies can enhance bee colony weight, mainly composed by honey, in later season when those native prairie forbs are blooming, suggesting an improvement in forage availability. To what extent honey bees would benefit from a more diverse community of flowering plants late in the season is not known. Furthermore, it remains to be tested whether honey bees in more diverse landscapes with more accessible floral resources are more efficient at foraging, requiring less energy spent in searching for pollen and nectar.

## Supplementary Material

toaa055_suppl_Supplemetary_Materials
