# Supplementary material for: Honey Bee (Hymenoptera: Apidea) Pollen Forage in a Highly Cultivated Agroecosystem: Limited Diet Diversity and Its Relationship to Virus Resistance
Source: J Econ Entomol. 2020 Apr 10;113(3):1062–72. doi: 10.1093/jee/toaa055 (PMC7362718; doi:10.1093/jee/toaa055)
Supplement: toaa055_suppl_Supplemetary_Materials [file toaa055_suppl_supplemetary_materials.docx]

**Supp. Table 1** Taxa of plants unidentified in the pollen collected by honey bees during 2015.

| Unidentified plant taxa (UT) ^§^ | % of each pollen type (Mean±SE) by weight | | Frequency* |
| --- | --- | --- | --- |
|  | High cultivation | Low cultivation |  |
| UT1 | 0.176±0.108 | 0.0856±0.0398 | 5 |
| UT2 | 0.1714±0.168 | 0 | 2 |
| UT3 | 0.1956±0.1839 | 0.1143±0.1032 | 4 |
| UT4 | 0 | 0.0523±0.0523 | 1 |
| UT5 | 0.1404±0.1404 | 0.0002±0.0002 | 2 |
| UT6 | 0 | 0.0319±0.0319 | 1 |
| UT7 | 0.0693±0.0693 | 0.0732±0.0732 | 2 |
| UT8 | 0.0082±0.0082 | 0 | 1 |
| UT9 | 0.0068±0.0068 | 0 | 1 |
| UT10 | 0.0014±0.0014 | 0.014±0.014 | 2 |
| UT11 | 0 | 0.003±0.003 | 1 |
| UT12 | 0.0004±0.0004 | 0.4961±0.4961 | 2 |
| UT13 | 0.0008±0.0008 | 0 | 1 |
| UT14 | 0.0166±0.0166 | 0 | 1 |
| UT15 | 0 | 0.0031±0.0031 | 1 |
| UT16 | 0 | 0.0003±0.0003 | 1 |
| UT17 | 0.012±0.012 | 0 | 1 |
| UT18 | 0.0197±0.0197 | 0 | 1 |

* Frequency was calculated as the number of sites at which a pollen type was found.

^§^ UT, unidentified taxa in our pollen collection.

**Supp. Table 2** Taxa of plants unidentified in the pollen collected by honey bees during 2016.

| Unidentified  plant taxa  (UT) ^§^ | % of each pollen type (Mean±SE) by weight | | Frequency* |
| --- | --- | --- | --- |
|  | High cultivation | Low cultivation |  |
| UT-3 | 0.501±0.2744 | 1.013±0.4093 | 8 |
| UN-2 | 0.0194±0.0156 | 0.0423±0.0423 | 3 |
| UN-13 | 0.0108±0.0108 | 0.0334±0.0255 | 3 |
| UT-19 | 0.0022±0.0022 | 0.0343±0.0343 | 2 |
| UT-20 | 0.0218±0.0207 | 0 | 2 |
| UT-21 | 0.0376±0.0376 | 0.0396±0.0396 | 2 |
| UT-22 | 0.0617±0.0427 | 0.0499±0.0499 | 3 |
| UT-23 | 0.0215±0.0215 | 0.0022±0.0022 | 2 |
| UT-24 | 0 | 0.0071±0.0071 | 1 |
| UT-25 | 0.0103±0.0103 | 0.0178±0.0178 | 2 |
| UT-26 | 0 | 0.0593±0.0593 | 1 |
| UT-27 | 0.2211±0.1397 | 0.0085±0.0085 | 3 |
| UT-28 | 0 | 0.0359±0.0359 | 1 |
| UT-29 | 0.0902±0.055 | 0 | 3 |
| UT-30 | 0.1605±0.1605 | 0.0646±0.0588 | 3 |
| UT-31 | 0.1026±0.0919 | 0.6399±0.6311 | 5 |
| UT-32 | 0.0488±0.0488 | 0.0471±0.0471 | 2 |
| UT-33 | 0 | 0.0033±0.0033 | 1 |
| UT-34 | 0 | 0 | 0 |
| UT-35 | 0.0101±0.0101 | 0.398±0.386 | 3 |
| UT-36 | 0.0252±0.0252 | 0 | 1 |
| UT-37 | 0.0021±0.0021 | 0 | 1 |
| UT-38 | 0 | 0.0009±0.0009 | 1 |
| UT-39 | 0.0352±0.0352 | 0.0489±0.0277 | 4 |
| UT-40 | 0 | 0.0263±0.0218 | 2 |
| UT-41 | 0 | 0.0076±0.0076 | 1 |
| UT-42 | 0 | 0.0905±0.0826 | 2 |
| UT-43 | 0.0559±0.0314 | 1.6572±1.4738 | 7 |
| UT-44 | 0.2371±0.1467 | 0.2267±0.1279 | 6 |
| UT-45 | 0 | 0 | 0 |
| UT-46 | 3.8165±3.3079 | 0.0256±0.0256 | 3 |
| UT-47 | 0.0702±0.0702 | 0 | 1 |
| UT-48 | 1.9748±1.3745 | 0.3886±0.2556 | 6 |
| UT-49 | 0.0059±0.0059 | 0 | 1 |
| UT-50 | 0.2279±0.2279 | 0.1717±0.1717 | 2 |
| UT-51 | 0 | 0 | 0 |
| UT-52 | 0 | 0.0268±0.0268 | 1 |
| UT-53 | 0 | 0.1476±0.1476 | 1 |
| UT-54 | 0.0109±0.0109 | 0.0206±0.0206 | 2 |
| UT-55 | 0 | 0.2423±0.2423 | 1 |
| UT-56 | 0 | 0.4409±0.4409 | 1 |

* Frequency was calculated as the number of sites at which a pollen type was found.

^§^ UT, unidentified taxa in our pollen collection.

| Identification | Year | Total Taxa | | Shared taxa^*^ | | Unshared taxa | Number of Taxa in two landscapes | | Cropland^**^ | | Grassland^**^ | | Woodland^**^ | | |
| --- | --- | --- | --- | --- | --- | --- | --- | --- | --- | --- | --- | --- | --- | --- | --- |
|  |  |  |  |  |  |  | High | Low | High | Low | High | Low | High | | Low |
| Total taxa | 2015 | | 33 | | 17 | 16 | 25 | 25 |  |  |  |  | |  |  |
| Identified taxa | 2015 | | 15 | | 11 | 4 | 12 | 14 | 8 | 8 | 4 | 6 | | 0 | 0 |
| Unidentified taxa | 2015 | | 18 | | 6 | 12 | 13 | 11 |  |  |  |  | |  |  |
| Total taxa | 2016 | | 64 | | 41 | 23 | 51 | 54 |  |  |  |  | |  |  |
| Identified taxa | 2016 | | 26 | | 21 | 5 | 25 | 22 | 17 | 12 | 7 | 9 | | 1 | 1 |
| Unidentified taxa | 2016 | | 38 | | 20 | 18 | 26 | 32 |  |  |  |  | |  |  |

**Supp. Table 3** A summary of number of plant taxa found in pollen collected by honey bees in 2015 and 2016.

^*^ Taxa shared between low vs high cultivation landscapes.

^**^ Number of taxa associated with potential land use types found in each of the two landscape categories.


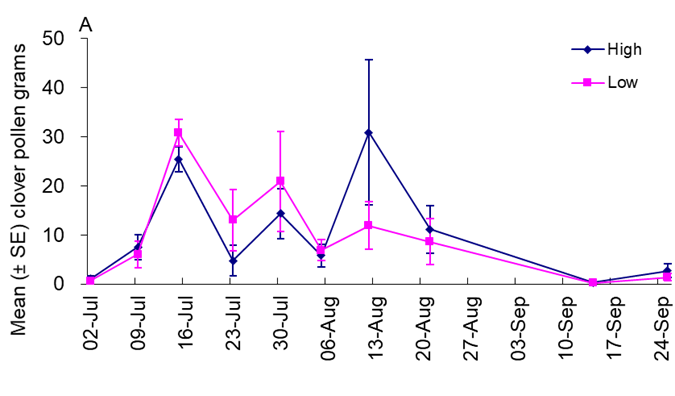


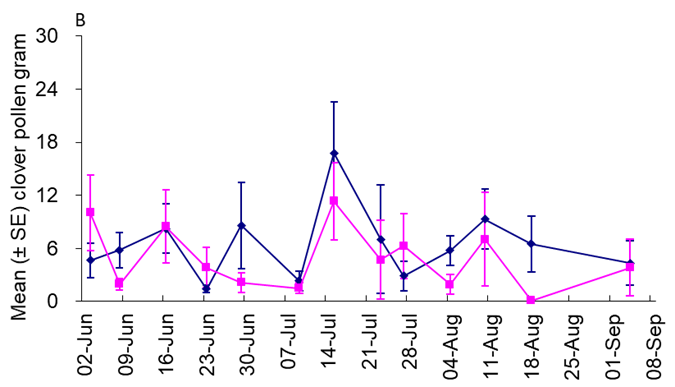


**Supp. Fig. 1** The amount of clover pollen collected by honey bees in two landscape classes in central Iowa during 2015 (A) and 2016 (B). There was no significant difference in the amount of pollen collected between the two landscape categories in both years (2015: F = 0.16, df = 1, 17.2, *P* = 0.695; F = 1.91, df = 1, 23.9, *P* = 0.180). The amount of pollen reported in the figure is based on the data without normalization by colony weight.


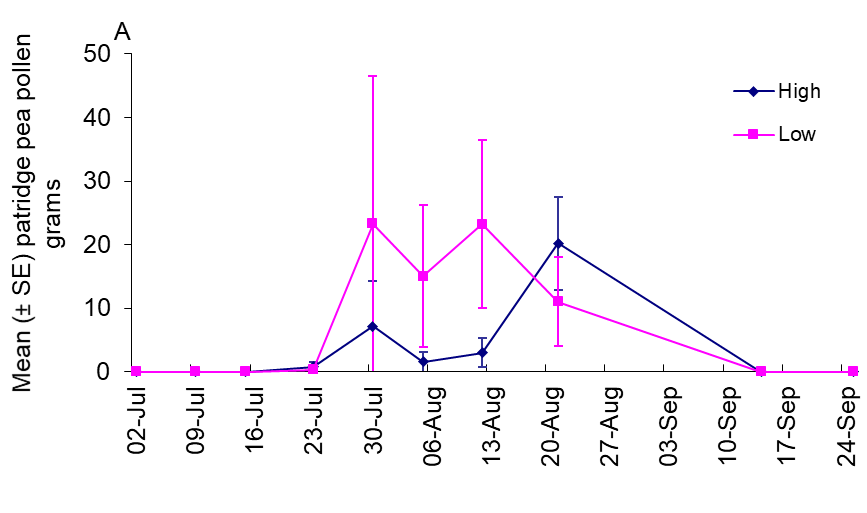


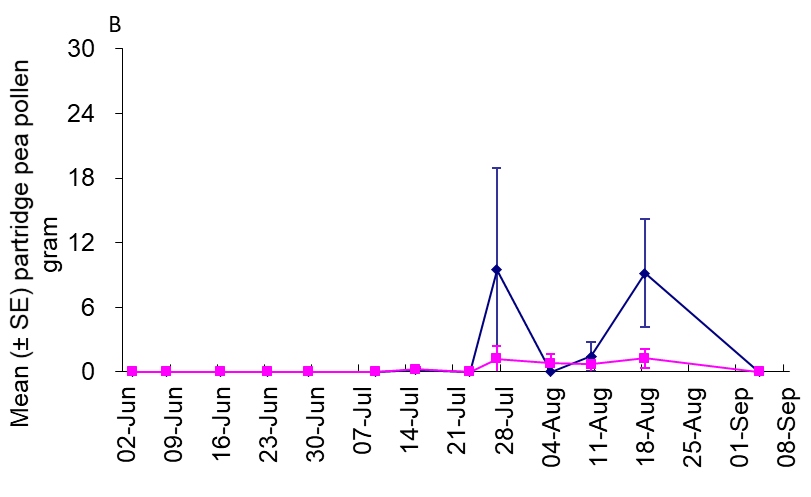


**Supp. Fig. 2** The amount of partridge pollen collected by honey bees in two landscape classes in central Iowa during 2015 (A) and 2016 (B). There was no significant difference in the amount of pollen collected between the two landscape classes in both years (2015: F = 0, df = 1, 18.7, *p* = 0.948; F = 0.16, df = 1, 17.2, *p* = 0.695). The amount of pollen reported in the figure is based on the data without normalization by colony weight.


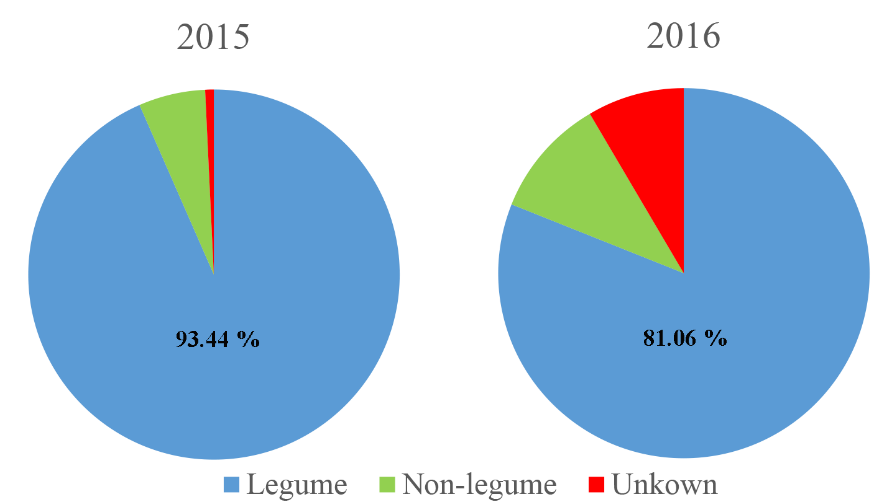


**Supp. Fig. 3** Contribution of legumes to pollen collection across all the landscapes in 2015 and 2016. Legumes included six plant taxa in both years: white clover (*Trifolium repens*), red clover (*Trifolium pratense*), partridge pea (*Chamaecrista fasciculata*)*,* birdsfoot trefoil (*Lotus corniculatus*), sweet clover (*Melilotus spp*.) and purple prairie clover (*Dalea purpurea*).
